# Is age‐related sarcopenia a real concern for my developing country?

**DOI:** 10.1002/jcsm.13107

**Published:** 2022-10-12

**Authors:** Arben Boshnjaku

**Affiliations:** ^1^ Physiotherapy Department, Faculty of Medicine University “Fehmi Agani” in Gjakova Gjakova Kosovo

## Abstract

Ageing is a distinctive feature of living organisms. With the modernization of human societies, including the development of science, technology and education, expected life expectancy at birth is being extended. This allows novel health‐related conditions to gain particular interest amongst the field experts. Along comes sarcopenia, an age‐related condition of global proportions that effects all populations, societies and countries. Several international working groups have been trying to identify the more appropriate and applicable sarcopenia definition and diagnostic criteria to follow. Contrary to the initial muscle mass‐related context, the shift of attention to muscle strength by the revised European Working Group in Sarcopenia for Older People (EWGSOP2) was ground‐breaking and potentially game‐changing. The suggested diagnostic algorithm by the EWGSOP2 for case finding, diagnosing and quantifying the severity of cases further facilitated the applicability on clinical practices.

Since being directly related to the ageing process, sarcopenia presents an issue of growing concern particularly within the high‐income and developed world regions that are generally characterized with an increased life expectancy. In contrast, the developing world and their generally lower life expectancy do not always have sarcopenia amongst the top targeted health‐related concerns. In such cases, the expected life expectancy and the populations' quality of life do not necessarily present an issue of major interest. Other serious medical concerns of acute state often eclipse the need for long‐term health‐related investments, shifting the interest towards only direct interventions and short‐term planning.

In conclusion, the emerging of sarcopenia as a serious age‐related concern is finding care providers and healthcare systems from lower and middle‐income countries (LMICs) unprepared. For the time being, it needs to be introduced and promoted in the developing world as a condition with direct life‐threatening implications. Simple and creative forms of approach should be ideated and implemented in both scientific and clinical contexts (by researchers and care providers, respectively). The best practice to address this situation would be by empowering intradisciplinary and interdisciplinary collaborations, as well as facilitating interconnections between researches, healthcare practitioners and clients. This should help establish sarcopenia as a serious age‐related condition that needs a multidisciplinary and multidimensional approach.

## Introduction

Researchers often like to discuss about intriguing topics and dare to address the ‘elephant in the room’ with no second thoughts. However, different individual or collective perspectives on a particular matter should not necessarily be seen in parity. Theoretical framework, as originating from inspiration and vision, take its proper meaning only when going hand by hand with the applicability in practice. After all, what started as a spark, blossomed as a condition, nowadays is even officially accepted as a muscle disease named sarcopenia that derives from a lifetime collection of adverse muscle changes.^1^

Much of the modern human's recent achievements, including the lower mortality rate at younger ages, higher longevity, better life quality and education in general, are attributed towards the societal organization and countries general level of development. Urbanization is amongst the distinctive features of major developed economies, whereas the urbanization rate itself has been described for a positive association with higher life expectancy.^2^ Ageing in general and age‐related conditions like sarcopenia in particular present global issues that affect all populations, notwithstanding the region or the level of development. Nevertheless, differences in this parameter are still observable in between the established developed and developing countries.

## What is the current consensus?

Several international working groups are constantly providing and updating suggestions on the better path to follow towards the more accurate, appropriate and accessible diagnostic screening for sarcopenia and its conceptual stages (pre‐/probable and severe). The shifting of attention for the major component from muscle mass as was the common understanding towards the muscle strength by the revised European Working Group in Sarcopenia for Older People (EWGSOP2)^1^ were seen as ground‐breaking and potentially game‐changing within the field. After all, the consensus working groups on definition and diagnosis aim to merge the gap in between research and clinical practice, thus easing the diagnostic process for this condition. Therefore, the diagnostic algorithm for case finding, diagnosing and quantifying the severity of cases as suggested by the EWGSOP2^1^ further facilitated the applicability on clinical practices. The Society for Sarcopenia, Cachexia and Wasting Disorders position paper^3^ further elucidated the process as a whole by supporting the diagnostic point of view and proceeding with recommended intervention strategies such as prescription of resistance exercise for any suspected case as well as increasing the individual protein intake of 1–1.5 g/kg/day in both primary and secondary sarcopenia.

## What is my developing countries position on the matter?

A developing country is typically characterized with lower industrial and human development index,^4^ representing the opposition of developed high‐income countries (HICs). In practice it is often provided as an inclusive term combining low‐income and middle‐income countries (LMICs), which together with the HICs present the three categories of per capita gross national income levels as defined by the World Bank.^5^ HICs and LMICs are characterized with an unequal distribution of relative life expectancy gains for older population, notwithstanding the substantial life expectancy gained by the global population in modern times.^6^ However, besides the currently lower percentages of older populations in LMICs, more than two‐thirds of this age‐group is expected to be living in LMICs by 2050,^7^ and might as well be considered as a serious demographic concern for a major shift of population structure in the future (e.g. the case of Kosovo where the population aged 65 and above is expected to increase from 8.1% in 2017 to 26.8% in 2061^8^). This brings sarcopenia to the equation as an ever‐growing concern with a global prevalence ranging between 10 and 27%.^9^ What worries most is not its current relatively high prevalence amongst older populations in HICs, but the prospect of rapid increase of populations that are directly affected by this condition within the LMICs. To date, most of sarcopenia‐related studies are conducted in populations from HICs of Caucasian background,^10^ with very few data being provided from LMICs.^11^ Nonetheless, higher prevalence of lower grip strength and gait speed^11^ as two out of three sarcopenia components and higher odds for fall‐related injuries amongst older adults^12^ have already been demonstrated in LMICs.

Another important point to be considered is that in a developing countries fragile healthcare system, expected life expectancy and the quality of life do not necessarily present an issue of major interest. Emergency medicine and acute medical interventions often shadow the needs for long‐term health investments and the development of appropriate strategies to target the needs. Recently, the ever‐growing phenomenon of antibiotic resistance and its association with high mortality risk particularly within the developing countries has been also brought to attention as a hot topic for such countries.^13^ Quite contrary, within a symptom‐oriented and disease‐targeted system as often observed in similar cases, preventive context of public health is rather undermined. In such circumstances, sarcopenia is not even an ‘elephant’ in a room that is filled with other ‘man‐eating and venomous creatures’. This directly affects older people with potential to develop age‐related diseases, because these non‐threatening conditions are often put on the backstage. However though, sarcopenia takes its natural course inside its habitat while causing progressive impairments and decline. When considering other implications such as the association with higher odds for fall‐related injuries,^12^ as well as its association with mild cognitive impairments amongst older people in LMIC's,^14^ it might just easily turn into unpredictable disaster for such societies. Even if not currently, it holds the potential to emerge as a serious concern for the near future.

The emerging of the new pandemics resulting from the novel SARS COVID‐19 virus infection did no good on the matter either, by digging further into the already fragile situation of these vulnerable groups of society. The outcomes are yet to be seen and count. On the other hand, when there are no regional or national requirements for professionals update towards a certain field, not everyone goes against the stream to do the opposite. Then, if the healthcare providers are not in the frontrunning of the fields' current state of art, it can easily create awkward situations such as being unable to provide the appropriate care for their patients. The awkwardness and misunderstandings might be increased with the influx of newer professionals when dealing with students and young healthcare experts as part of their clinical placements who are already taught about such issues within academia. The potential growing mistrust might lead towards lower professional cooperation and inter‐professional trust, always being translated into declining care for the final end users. However though, these new generations represent the future frontrunners in the fight against an issue that is expected to gain prominence amongst the ageing problems of LMICs. Thus following the current trends, chances are that even if not systemically, many developing countries will at least have some human resources available for the future battles against this devastating disease.

## How to approach this issue and future directions?

Summarizing the facts, sarcopenia is emerging as a serious age‐related concern that is currently finding care providers from LMICs unprepared and in the lack of resources to be dealt with. To address this situation, several steps are needed to be applied.

The first and foremost path to follow would be to merge the needs and facilitate the connections in between the researchers that develop, clinicians that provide and end users that receive the goods. It must start with further exemplifying the guidelines and spreading the word in a simplified and realistic manner. Besides being assigned with an ICD‐10‐CM code (M62.84) in 2016 and warmly accepted by the major stakeholders in the field,^15^, ^16^ the need to be further emphasized and promoted seems inevitable for sarcopenia.

**Figure 1:**
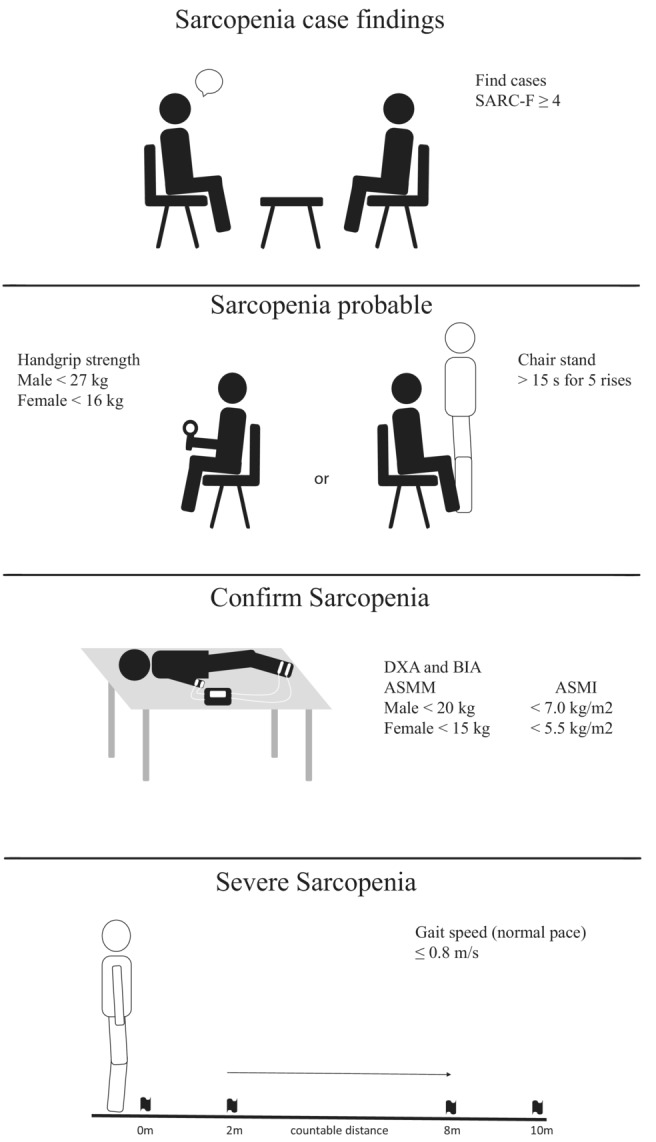
An informative step‐by‐step diagnostic process following the suggested algorithm and diagnostic criteria as set by the revised European Working Group on Sarcopenia in Older People.^1^

The SARC‐F questionnaire is the very first instrument that is used as a screening tool for case findings in practice, based on five different components: strength, walking assistance, chair rise, stair climb and falls.^17^ As shown in *Figure* 1, a simple A3 format poster with an illustrated step‐by‐step diagnostic process that would be placed in every examination room within the primary healthcare providing centres including the SARC‐F, isometric handgrip strength assessment and/or 30‐s chair stand test protocol would be enough to have a probable sarcopenia diagnosis. These cases then would be referred for sarcopenia diagnostic confirmation through bioelectrical impedance analysis (BIA), dual‐energy X‐ray absorptiometry (DEXA), magnetic resonance imaging (MRI) or computerized tomography (CT), as well as the sarcopenia severity through physical performance assessment (gait speed of usual walk).

A final pathway to follow would be to emphasize the nutritional impact and benefits from planned diets on the sarcopenia management process, particularly because the nutritional habits and food insecurity have been shown to be associated with an increased risk for sarcopenia in LMICs.^18^ Furthermore, it should be important to provide and enable management strategies, at least to give practitioners the feeling of their power to handle such situations. It should not be forgotten that even though presenting an issue of increasing interest amongst researchers and clinicians around the world, sarcopenia might just not be amongst the top healthcare priorities in the developing world. It is obvious that fields like acute medicine, intra‐hospital infections and antibiotic resistance are undoubtedly priority within the developing world. Therefore, it is of great importance to promote sarcopenia within the developing world as a novel condition with direct life‐threatening implications.

## Conflict of interest

The author declares no conflict of interest.
